# Supplementary material for: Content-rich biological network constructed by mining PubMed abstracts
Source: BMC Bioinformatics. 2004 Oct 8;5:147. doi: 10.1186/1471-2105-5-147 (PMC528731; doi:10.1186/1471-2105-5-147)
Supplement: Additional File 2 — The original results of the above study (non-essential files are deleted to keep the file size under the limit set by BMC bioinformatics). [file 1471-2105-5-147-S2.bz2 › chilibotAdditionalFile2/dip30/279ID2498083E1102/html/FOSB_JUN.html]

 


 **FOSB** and **JUN** 
  
Found 189 abstracts in PubMed, retrieved 30.  
 

 What does Google say? 
 PDF only 
| .edu only 

---

**Interactive relationship** (e.g. stimulation, inhibition, etc)

**Neutral relationship**- Based on in vitro analyses, GnRH appeared to enhance the interaction of JunD,  **FosB** , and c Fos at the GnRHR AP 1  [ **JUN** ]  element.  Ref: 12586760 Endocrinology, 2003
- All cells expressed fos, fra1 and  **jun**  proteins and none expressed  **fosB** .  Ref: 11809534 Cancer Lett, 2002
- **FosB**  is a member of the AP 1  [ **JUN** ]  family of transcription factors which represent important regulators of cell proliferation and differentiation.  Ref: 12602926 Breast Cancer Res Treat, 2003
- **fosB**  was up regulated 20 fold, but only minor effects on  **jun**  variants were observed.  Ref: 12820376 Anticancer Res< MedlineTA>Anticancer Res, 2003
- Under conditions of high rhodopsin availability during exposure to light, Fra 2 and, to a minor degree,  **FosB**  substitute for c Fos and enable light induced AP 1  [ **JUN** ]  activity and thus photoreceptor apoptosis.  Ref: 12766089 Invest Ophthalmol Vis Sci, 2003
- Phosphorylated c  **Jun** , JunB, JunD, and Fra 1, but not c Fos,  **FosB** , or ATF 2, are detected in the AP 1  [ **JUN** ]  DNA binding complex in cells exposed to trivalent arsenicals.  Ref: 12475910 FASEB J, 2003
- These studies demonstrate that a functional AP 1  [ **JUN** ]  site mediates MMP 2 transcription in cardiac cells through the binding of distinctive Fra1 JunB and  **FosB**  JunB heterodimers.  Ref: 12371906 Biochem J, 2003
- Gene expression and nuclear translocation of all the AP 1  [ **JUN** ]  members were induced, under both conditions, with differences in their kinetics, except  **fosB**  mRNA in the Clinostat.  Ref: 12020768 Cell Signal, 2002
- Forty six globes were processed for in situ hybridization with oligonucleotide probes for c fos,  **fosB** , c  **jun** , junB and junD mRNAs, and 60 globes were immunohistochemically analysed using anti c Fos and anti c  **Jun**  antibodies.  Ref: 11825018 Exp Eye Res, 2001
- Here we show that transcriptional activation of an AP1  [ **JUN** ]  regulated gene through the mitogen activated protein kinase extracellular signal regulated kinase pathway is characterized, in intact cells, by a switch from a fra2 junD dimer to a junD  **fosB**  dimer loading on its promoter and by simultaneous recruitment of ERKs, CREB binding protein, and RNA polymerase II.  Ref: 12052862 Mol Cell Biol, 2002
- The Wnt mediated repression of c Fos,  **FosB** , and JunB expression was consistent with a decrease in their binding to an AP 1  [ **JUN** ]  promoter element and decreased target gene transcription.  Ref: 11751871 J Biol Chem, 2002
- Electrophoretic mobility shift assays using nuclear extracts of Jurkat T cells as well as TCR CD3 restimulated primary human T cells demonstrated specific binding of AP 1  [ **JUN** ] , predominantly composed of c  **Jun**  and  **FosB** , to this sequence.  Ref: 12618758 Oncogene, 2003
- Fra 1, Fra 2,  **FosB** , JunB, JunD, c  **Jun** , and c Fos levels are increased by EGCG treatment, as is AP1  [ **JUN** ]  factor binding to hINV promoter AP1  [ **JUN** ]  site.  Ref: 11698415 J Biol Chem, 2002

**Non-interactive relationship** (e.g. studied together, co-existance, homology, etc.)

- We have investigated the expression of the AP 1  [ **JUN** ]  transcription factor proteins, fos,  **fosB** , fra1, fra2,  **jun** , junB, junD, using Western blot analysis, in several types of asynchronously proliferating cells.  Ref: 11809534 Cancer Lett, 2002
- Ectopic expression of transdominant negative  **Jun**  mutants strongly reduced CD95L promoter activity and activation induced cell death, confirming the functional significance of  **FosB**  c  **Jun**  binding.  Ref: 12618758 Oncogene, 2003
- Using DNA protein gel shift assays we determined that nerve growth factor stimulates AP 1  [ **JUN** ]  binding in both PC12 and B5P cells, and identified c Fos,  **FosB** , Fra 1, Fra 2, c  **Jun** , JunB and JunD in AP 1  [ **JUN** ]  complexes.  Ref: 11905996 J Neurochem, 2002
- Immunoblot analyses confirmed that c  **Jun** , JunD, JunB, c Fos, Fra 1, Fra 2 and  **FosB**  immunoreactive proteins were present in whole cell extracts of all antral follicles and midluteal phase corpora lutea as well as granulosa cells isolated from different sized antral follicles.  Ref: 12606371 Biol Reprod< MedlineTA>Biol Reprod, 2002
- Among the early response genes analyzed, c myc, junB, junD, c  **jun** , c fos,  **fosB** , fra, as well as max, mad1 4, sin3, only c  **jun**  and fra 2 mRNAs were up regulated after 5D exposure.  Ref: 12648525 J Steroid Biochem Mol Biol, 2003
- We further studied the expression of  **fosB** , c  **jun**  and junB, in the AP 1  [ **JUN** ]  complex.  Ref: 12820376 Anticancer Res< MedlineTA>Anticancer Res, 2003
- This effect was specific because palytoxin had little effect on c  **Jun** , JunB, JunD,  **FosB** , Fra 1, or Fra 2 binding or on overall levels of transcription factor binding.  Ref: 12460732 Toxicol Appl Pharmacol, 2002
- In primordial and primary unilaminar follicles, all AP 1  [ **JUN** ]  factors studied except for  **FosB**  were detected in granulosa nuclei.  Ref: 12606371 Biol Reprod< MedlineTA>Biol Reprod, 2002
- but there was a marked induction of  **FosB**  and Krox 20, a strong repression of c  **Jun** , and no effect or a reduction of the basal levels of JunD.  Ref: 11744254 Neuroscience, 2001
- Thus, our results demonstrate an important novel function for  **FosB**  dimerized with c  **Jun**  in TCR CD3 mediated AICD in human T cells.Oncogene 2 333 1339.  Ref: 12618758 Oncogene, 2003
- In contrast, the AP1  [ **JUN** ]  site mediates an osteoblast specific activation caused by the preferential binding of  **FosB**  to CE1 in osteoblastic cells.  Ref: 12186862 J Biol Chem, 2002
- Within the nucleus, Nrf1 and Nrf2 are recruited to the ARE as heterodimers with either small Maf proteins,  **FosB** , c  **Jun** , JunD, activating transcription factor 2 ATF2 or ATF4.  Ref: 11689285 Cancer Lett, 2001
- The defect is secondary to an inability to phosphorylate CREB and to induce CREB dependent immediate early genes, including c  **jun** ,  **fosB** , fra2, and junB, which are required for cytokine gene induction.  Ref: 11739719 Mol Cell Biol, 2002
- Normal lens epithelial cells expressed mRNA signals for junD, but not for c fos,  **fosB** , c  **jun** , and junB. mRNAs for c fos,  **fosB** , c  **jun** , and junB were detected in the whole lens epithelium from the vicinity to the wound to the equator from 30 min to 8 hr post injury with their peaks after 30 min to 1 hr, but were no longer detected at 10 hr or later.  Ref: 11825018 Exp Eye Res, 2001
- Because these two proteins did not give any detectable FRET uponantigen addition, we tethered each protein with a leucine zipper motif c  **Jun**  or  **FosB**  at the C terminus to help the neighborhood of the GFP variants.  Ref: 12463362 Anal Chem, 2002
- In the lateral thalamus the repeated stimulation again failed to induce c Fos, but the expressions of  **FosB** , c  **Jun**  and Krox 24 were increased,.  Ref: 11744254 Neuroscience, 2001
- Of note, in OA chondrocytes, IL 17 and IL 1beta induced collagenase 3 production through AP 1  [ **JUN** ]  occurred with differential protein complexes IL 17 stimulation resulted in  **FosB**  activation, while IL 1beta stimulated c Fos.  Ref: 12064845 J Rheumatol, 2002
